# Therapeutic targets in diabetic peripheral neuropathy: heat shock proteins

**DOI:** 10.3389/fendo.2025.1729488

**Published:** 2025-12-18

**Authors:** Chengzhi Yin, Jiao Lv, Shujia Huang, Chenyu Lang, Yunyun Zhao, Guoqiang Wang, Junming Kan, Xiuge Wang

**Affiliations:** 1 College of Chinese Medicine, Changchun University of Chinese Medicine, Changchun, Jilin, China; 2 College of Basic Medicine, Changchun University of Chinese Medicine, Changchun, Jilin, China; 3 Endocrinology Department, First Affiliated Hospital to Changchun University of Chinese Medicine, Changchun, Jilin, China

**Keywords:** diabetic peripheral neuropathy, heat shock protein, molecular chaperone, nerve, protein folding

## Abstract

Diabetic peripheral neuropathy (DPN), a debilitating diabetic complication, has a complex pathological mechanism involving oxidative stress, mitochondrial dysfunction, and endoplasmic reticulum stress, and there are no effective disease-mitigating treatments. Current management is restricted to glycaemic control and symptomatic analgesia, both of which offer only modest benefit and carry appreciable adverse-effect profiles. Heat Shock Proteins (HSP) are stress-inducible chaperones that counteract protein misfolding and aggregation. Through suppression of apoptosis, cytoskeletal stabilisation and immune modulation they exert neuroprotective effects relevant to DPN onset and progression. Studies have shown that HSP90 regulates neuronal plasticity and that its inhibitors restore mitochondrial function in diabetic neurons, whereas HSP70 and HSP27 exert context-dependent positive or negative regulation. Subsequent work has evaluated covalent HSP90 inhibitors, novel HSP70 agonists, Trans-activator of transduction-Heat shock protein 27 (TAT-HSP27) mediates suppression of mitochondrial apoptosis and the utility of HSP27 as a circulating biomarker. Here we synthesise recent advances in HSPs biology and DPN pathogenesis, highlight the therapeutic potential of targeting HSPs and outline translational strategies that may expedite disease-modifying therapy.

## 1 Introduction

Diabetic peripheral neuropathy (DPN) is the most frequent chronic complication of diabetes, affecting approximately 50% of patients with type 1 (T1DM) or type 2 (T2DM) diabetes during their lifetime. In China, the age-adjusted prevalence of DPN has been reported at 21.9% in T1DM and 35.3% in T2DM (1–3). DPN manifests as numbness or neuropathic pain and may progress to foot ulceration and amputation; it carries a 3.6-fold higher risk of major amputation and a 10-fold higher risk of peripheral arterial disease, severely impairing quality of life (4, 5). DPN arises from multiple interconnected pathological mechanisms. These include disturbances in insulin signalling, accumulation of advanced glycosylation end products (AGEs), and abnormal polyol metabolism. Oxidative stress and inflammatory responses further contribute to disease progression (6–10). Pharmacologically, drugs such as proton pump inhibitors and metformin may worsen DPN through vitamin B12 deficiency (11, 12). Current clinical management emphasizes strict glycaemic control, cardiovascular risk reduction, and symptomatic pain relief. However, no FDA-approved therapy currently targets the core pathophysiology of DPN (2).Pharmacological treatment of neuropathic pain in DPN relies primarily on anticonvulsants (e.g., pregabalin, gabapentin) and antidepressants (e.g., duloxetine, amitriptyline) (13–16). Although opioids can reduce neuropathic pain intensity, their high potential for addiction, abuse liability and dose-limiting adverse events (e.g., sedation, somnolence, headache and gastrointestinal hypomotility) restrict long-term use. Consequently, current guidelines advise against opioid therapy for painful diabetic peripheral neuropathy on the basis of an unfavourable risk–benefit ratio (2). Given the limited efficacy of existing treatments for DPN and neuropathic pain, the need for additional adjunctive therapies remains unmet. In the management of DPN, the use of nutritional supplements can actively intervene in the underlying neuropathological mechanisms and improve clinical prognosis beyond the relief of symptomatic pain, which is often the ultimate goal of existing analgesic therapies (17).

**Figure 1:**
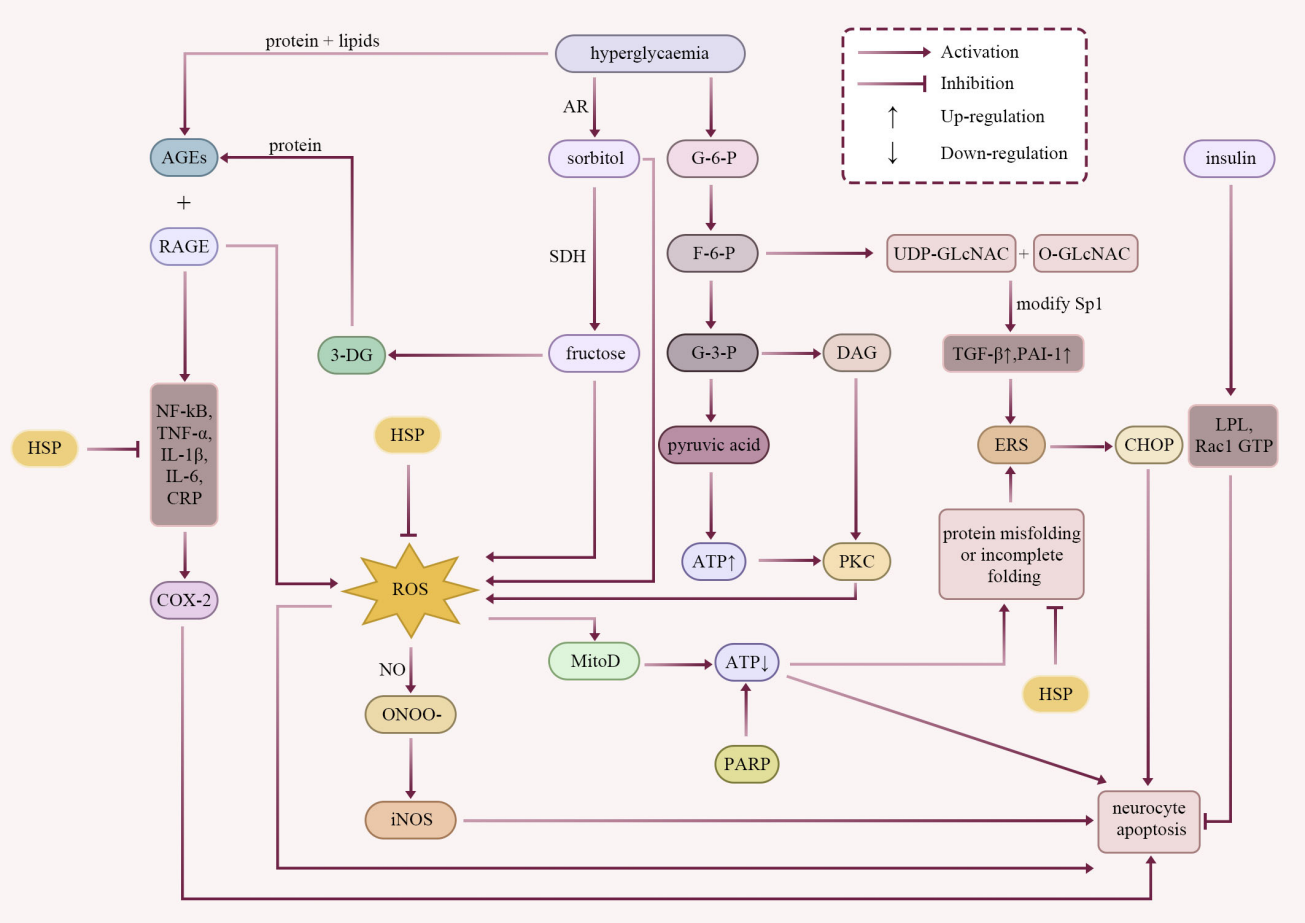
Pathogenesis of DPN. Current research into the pathogenesis of DPN indicates that insulin suppresses neuronal apoptosis under chronic hyperglycaemic conditions, while multiple interrelated mechanisms collectively promote the onset and progression of DPN. These pathological processes predominantly promote the expression of inflammatory cytokines, thereby triggering inflammatory responses (e.g., NF-kB, TNF-α, IL-1β, IL-6, CRP). Additionally, they induce ROS production, exacerbating oxidative stress and disrupting protein homeostasis. Ultimately, this leads to neuronal cell apoptosis. HSPs constitute the first line of defence against protein misfolding and the accumulation of aggregation-prone proteins. HSPs can suppress the expression of multiple inflammatory cytokines and mitigate oxidative stress responses. In summary, HSPs exert crucial regulatory roles in the pathogenesis and progression of DPN, offering novel therapeutic targets for intervention strategies.

The pathogenesis of DPN has not been fully elucidated, and the pathological process involves activation of the polyol pathway, accumulation of AGEs, nitrosative stress-related cytotoxicity, endoplasmic reticulum stress (ERS), mitochondrial dysfunction, protein kinase C (PKC) signalling pathway, hexosamine pathway, poly ADP-ribose polymerase (PARP) pathway, dyslipidaemia, inflammatory response, disruption in insulin signalling pathway, and other multiple interrelated factors (**Figure 1**).

Chronic hyperglycaemia drives the onset and progression of DPN through multiple interrelated pathways. A key mechanism is the sustained upregulation of the polyol pathway. In this process, aldose reductase (AR) converts glucose to sorbitol, which is then oxidized to fructose by sorbitol dehydrogenase (SDH). The intracellular accumulation of these polyols induces osmotic stress and contributes to neural injury. Concurrently, the consumption of reduced coenzyme II (NADPH) is diminished, thereby impairing endogenous antioxidant defences and further amplifying oxidative stress (18, 19). Concurrently, hyperglycaemia contributes to the formation and deposition of AGEs in neural tissues, modifying key proteins and disrupting neural structures. AGEs induce large amounts of reactive oxygen species (ROS) production by binding to the receptor RAGE (Advanced glycosylation end products receptor). And activating inflammatory pathways such as NF-kB (mammalian transcription factor NF-kB family pathway), interleukin 6 (IL-6), IL-1β, and TNF-α (tumour necrosis factor-alpha), which further lead to oxidative damage and apoptotic (20, 21). Notably,3-Deoxyglucuronide (3-DG) produced in the polyol pathway also promotes AGEs production, creating a vicious cycle (22). Concurrently, nitrative stress is a key driver of early DPN through formation of peroxynitrite (ONOO^-^) from superoxide and nitric oxide (NO), which directly injures neural structures and amplifies injury by up-regulating inducible nitric oxide synthase (iNOS) (23). Accumulation of misfolded proteins triggers ERS, leading to neuronal apoptosis. One key pathway is the IRE1α-XBP1 (inositol-requiring enzyme 1α-X box-binding protein 1) axis. The activation of this axis upregulates the pro-apoptotic transcription factor CHOP (C/EBP homologous protein), which ultimately mediates the apoptotic cell death (24–26). Mitochondrial dysfunction is a central driver of hyperglycaemic neuroenergetic impairment. This process is characterized by excessive ROS production, which impairs the electron transport chain and reduces adenosine triphosphate (ATP) synthesis. The resulting energy deficit preferentially injures distal small-calibre nerve fibres. This energy deficit provokes neuropathic pain and sensory dysfunction. Schwann cells (SC) provide robust support to large myelinated fibres but offer limited protection to small fibres, rendering the latter particularly vulnerable to injury (2, 27–29). Further, glycolysis as a major pathway of glucose metabolism, in which the accumulation of the intermediate product diacylglycerol (DAG) promotes over-activation of PKC signalling. This causes glial cell dysfunction, reduced glutamate uptake and increased ROS (30–32). Additionally, the hexosamine pathway modifies specificity protein 1 (Sp1) via O-β-D-N-acetylglucosamine (O-GlcNAc), thereby up-regulating transforming growth factor-β (TGF-β) and plasminogen activator inhibitor-1 (PAI-1), which amplifies ERS and oxidative damage (33–38). In DPN, PARP hyperactivation consumes nicotinamide adenine dinucleotide (NAD^+^), precipitating an energy crisis and amplifying oxidative/nitrative stress and inflammation (39–41). In addition to glucose metabolism, abnormalities in lipid metabolism independently exacerbate nerve damage, and lipid peroxidation products are neurotoxic (42–46). Chronic, low-grade inflammation is a persistent feature of DPN. This state is characterized by elevated levels of systemic markers, including C-reactive protein (CRP), IL-6, and soluble intercellular adhesion molecule-1 (sICAM-1), alongside the up-regulation of cyclooxygenase-2 (COX-2). Together, these factors amplify local neuroinflammation and contribute to neuropathic pain (47–50). Finally, abnormal insulin signalling directly impairs myelin protein expression and hinders axon regeneration. Moreover, insulin exerts neurotrophic and reparative effects through key mediators, including lipoprotein lipase (LPL), Ras-related C3 botulinum toxin substrate 1 (Rac1 GTP) and PKC pathways (15, 51–53).

Heat Shock Protein (HSP) is a class of highly structurally conserved peptides widely distributed in prokaryotic and eukaryotic organisms, which is mainly involved in the repair process after cellular damage and is an important component of protein stability. Since the 1970s, HSP synthesis has been shown to be inducible by diverse stressors. Including hypothermia, heavy metals, nutrient deprivation, hypoxia and heat. So they are also termed stress proteins (SP) (54). HSP is the first line of defence against the accumulation of misfolded, aggregation-prone proteins. Its neuroprotective effects include inhibition of apoptosis, cytoskeletal protection and immunomodulation.

The pathogenesis of DPN is highly complex and multiple stress responses are involved. Therefore, future studies should explore more deeply the regulatory role of cytoprotective mechanisms in the stress responses including HSP in the occurrence and development of DPN, and provide a new direction for multi-targeted integrated intervention strategies.

## 2 Heat shock proteins

**Figure 2:**
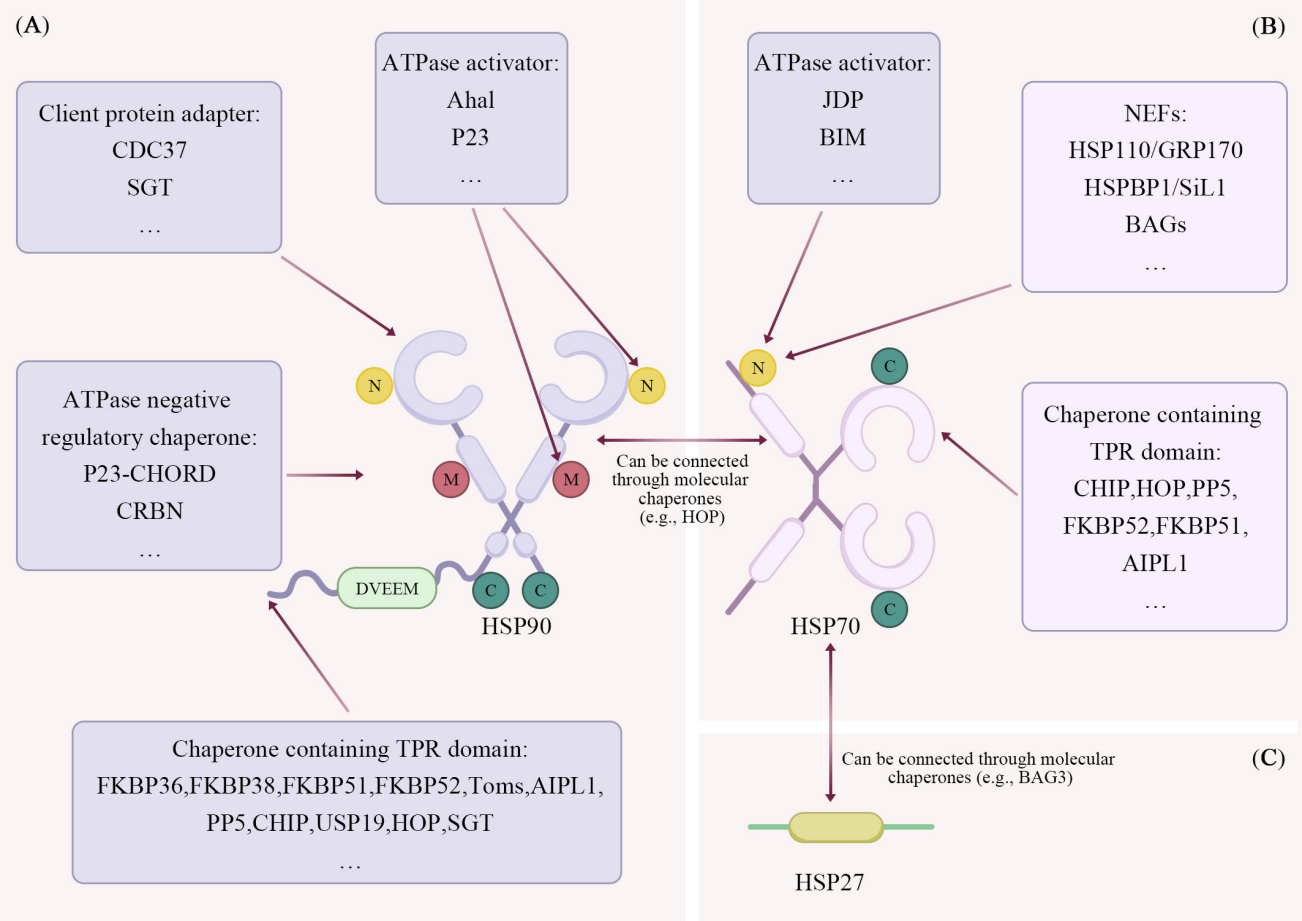
Structural and functional characteristics of HSP90, HSP70 and HSP27. **(A)** HSP90 comprises three domains: NTD, CTD and MD. The NTD primarily mediates HSP90’s interaction with molecular chaperones and ATPase activators, thereby facilitating the reception and regulation of client protein folding and maturation. The CTD predominantly binds co-chaperone proteins containing TPR domains via its MEEVD motif, extensively participating in client protein regulation. The MD is responsible for binding ATPase activators and inhibitors, thereby maintaining stable client protein binding states and balancing the effects of activators and inhibitors on client proteins. **(B)** HSP70 comprises two domains: the NBD and the SBD. The NBD regulates client protein binding and release by interacting with ATPase activators and NEFs. The SBD binds to co-chaperone proteins containing TPR domains, regulating protein folding through its switchable peptide-binding cavity. Additionally, HSP70 and HSP90 can interact via HOP, which facilitates the transfer of client proteins from HSP70 to HSP90. This process promotes the maturation of client proteins. **(C)** HSP27 is a small-molecule heat shock protein lacking enzymatic activity and is also ATP-independent. It functions both as the primary defence mechanism for intracellular protein homeostasis and interacts with other chaperone proteins to regulate protein stability. Furthermore, HSP27 and HSP70 can bind via BAG3 to jointly regulate protein homeostasis.

According to molecular weight, HSP can be classified into subtypes such as HSP100, HSP90, HSP70, HSP60, HSP40 and small molecule HSP families (e.g. HSP27) (55). Among them, large molecule HSPs, such as HSP90, HSP70, and HSP60, require dynamic changes in their three-dimensional conformation and hydrolysis of ATP to exert their chaperone activity. Small molecule HSPs are typical ATP-independent molecular chaperones, which are usually considered as passive retention enzymes. In animal models, HSP is one of the most potent inhibitors of neurodegeneration. Thus, HSP provides a potential target for protective drug therapy in many neurological diseases (56). Here, we focus on the three best-characterised and the most described chaperones—HSP90, HSP70 and HSP27 (**Figure 2**).

### 2.1 HSP90

Cytoplasmic HSP90 is constitutively abundant and further inducible by cellular stress. Its main role is to interact with a specific set of proteins in their natural or near-natural state in order to promote the maturation of their conformation. These proteins include transcription factors, transduction molecules, tyrosine kinases, serine/threonine kinases (57–61). HSP90 consists of three structural domains: the N-terminal structural domain (NTD), the C-terminal structural domain (CTD) and the middle structural domain (MD) (62). NTD mediates the interaction of ATP with HSP90 and conformational changes in HSP90 (63, 64). The CTD contains a calmodulin binding site, a homodimerisation motif and a nucleotide binding site that functions as a variable regulator of NTD ATPase activity (65). The major isoforms of mammalian HSP90 include cytoplasmic HSP90 α and HSP90 β, endoplasmic reticulum glucose-regulated protein-94 (GRP94) and mitochondrial TNF receptor-associated protein 1 (TRAP1) (66–68).

HSP90 molecular chaperones regulate the folding and maturation of client proteins through their ATPase cycle, a process that is dynamically regulated by multiple co-chaperones. Firstly, in the pre-regulatory phase of HSP90, cell division cycle protein 37 (CDC37) facilitates the binding of HSP90 by specifically recruiting kinase-like clients (e.g., the class I serine/threonine kinase RAF and the cytosolic protein-dependent kinase CDK) (69, 70). Subsequently, the mechanism by which the ATPase activator Aha1 (Activator of hsp90 ATPase activity 1, also known as AHSA1/p38) binds to HSP90 involves its N-terminal domain docking onto the mesomolecular domain of HSP90. Whereas its CTD transiently binds the HSP90 NTD, promoting N-terminal dimerisation and accelerating ATP hydrolysis. This Aha1-driven stimulation of the HSP90 ATPase powers the late-stage folding of the client protein (71). However, the ATPase activity of HSP90 is also negatively regulated in several ways. For example, Prostaglandin E synthase 3 (P23) binds HSP90 MD through its folded cysteine- and histidine-rich structural domain (CHORD) during the late stages of HSP90 regulation, stabilising the closed conformation of HSP90 and reducing ATPase activity by approximately 50%, thereby maintaining the stable binding state of the client protein (72). Similarly, the E3 ubiquitin ligase component cereblon (CRBN) inhibits ATPase activity and antagonises the negative effects of Aha1 on client protein stability by specifically recognising the MD of the HSP90 dimer in the ATP-bound state (73). In addition, multiple TPR domain-containing co-chaperones (a class of highly conserved protein structural domains capable of forming α-helix bundles that bind to other proteins or nucleic acids, also known as Tetratricopeptide Repeat) function by recognising the Met-Glu-Glu-Val-Asp motif (MEEVD) of the HSP90 CTD. The family of FK506-binding proteins (e.g. FKBP36/38/51/52) Bind to HSP90 through the TPR structural domain and are extensively involved in the regulation of client proteins (74, 75). Mitochondrial outer membrane translocase (Toms) uses TPR motifs to interact with HSP90, assisting in the import of pre-mitochondrial proteins and preventing their aggregation in the cytoplasm (76–80). Protein phosphatase 5 (PP5) is also activated by HSP90 through a similar mechanism, which in turn dephosphorylates specific substrates (81). In terms of fate determination of client proteins, the heat shock homologous protein 70 (HSC 70) interacting protein (CHIP, also known as CTD HSC70-responsive protein) acts as a ubiquitin ligase and interacts with HSP90 through the TPR and U-box structural domains to ubiquitinate client proteins and direct them to proteasomal degradation, as well as triggering the release of P23 and the remodelling of HSP90 complexes (82–84). In contrast, the deubiquitinating enzyme, ubiquitin-specific protease 19 (USP19), promotes substrate stability by binding to HSP90 (85). Finally, HSP70 and HSP90 histones (HOP) and glutamine-rich small TPR proteins (SGT) act as splice proteins to coordinate collaboration between HSP70 and HSP90. HOP recognises HSP90 and HSP70 CTDs through its TPR2A and TPR2B structural domains, respectively, to form a ternary complex that assists in the transfer of clients from HSP70 to HSP90 and facilitate their maturation process (86–90). SGT also mediates both interactions, providing a platform for substrate loading (91, 92).

### 2.2 HSP70

HSP70 is ubiquitous across organelles, where it catalyses nascent-polypeptide folding, refolds misfolded proteins, drives membrane translocation and directs irreversibly damaged clients to degradation (93, 94). HSP70 contains two structural domains: an N-terminal nucleotide-binding structural domain (NBD), a C-terminal substrate-binding structural domain (SBD) (95). The NBD fuels chaperone activity by ATP binding and hydrolysis. The SBD is subdivided into a substrate-binding domain (SBDβ) and a helix-capping domain (SBDα) that together create the peptide-binding cavity. In the nucleotide-free or ADP-bound state, the SBDα docks onto SBDβ, sealing the substrate-binding cavity. This closed conformation allows HSP70 to effectively recognize, bind, and fold nascent or misfolded polypeptides (96). Human cell contain a number of HSP70 family members, including HSC 70, stress-inducible HSP70, mitochondrial glucose-regulated protein-78 (GRP78) and endoplasmic reticulum glucose-regulated protein-75 (GRP75). Basal ATPase activity of these paralogues is low but is markedly stimulated by interaction with J-domain proteins (JDP) of the HSP40 family (57, 58, 97). Some macromolecular HSPs also act mainly as auxiliary molecular chaperones for HSP70 (98, 99).

B-cell lymphoma-2 (Bcl-2) interaction mediator (BIM, an important apoptosis-regulating protein), a Bcl-2 family protein, also functions as an auxiliary chaperone for HSP70. It binds to the HSP70 NBD via its Bcl-2 homology 3 (BH3) domain, thereby enhancing HSP70 ATPase activity and modulating its conformational state. This interaction ultimately stabilizes the binding of HSP70 to its client proteins (100, 101). The ATPase cycle of HSP70 begins with the hydrolysis of ATP to generate adenosine diphosphate (ADP) and free phosphate group (Pi), which in turn causes a conformational change in the SBD prompting substrate release. Under physiological conditions the rate of dissociation of ADP is the key rate-limiting step for substrate release (102). In this process, nucleotide exchange factors (NEFs), such as HSP110/GRP170, HSPBP1/Sil1 and BAG (Bcl-2-associated athanogene) family members, promote the opening of the cleft between structural domains by interacting with the HSP70 NBD, accelerating the release of ADP and ultimately leading to the dissociation of the substrate from SBDs (96, 102). In addition, a variety of TPR structural domain-containing co-chaperones also play an important role in the regulation of HSP70 function. For example, CHIP inhibits HSP40-activated HSP70 ATPase activity and uses its E3 ubiquitin ligase activity to degrade misfolded proteins. HOP assists in the functional coupling of HSP70 to HSP90 and PP5 promotes the dephosphorylation of HSP70 substrate (86, 103–105). Other TPR proteins such as FKBP51/52 and aryl hydration carbon receptor interacting protein 1 (AIPL1) have also been reported to interact with HSP70. Although FKBP51/52 preferred to bind HSP90. Whereas HOP and CHIP showed a moderate preference for HSP70 (106, 107).

### 2.3 Small molecule HSP

Small molecule HSPs are ATP-independent, so they can act as a first line of defence for protein homeostasis in the cell (108). Currently, a total of 10 small molecule HSPs (HSPB 1-HSPB 10) have been identified in mammals, ranging in size from 12–43 kDa (109). Three small molecule HSPs associated with human disease include HSPαB-crystallin (HSPB 5), HSP27 (HSPB1), and HSP22 (HSPB8), largely distributed in the nervous system (56). The small molecule HSP can be divided into three structural parts: the variable NTD, the conserved α-crystallin structural domain, and the short CTD (110, 111). The α-crystallin structural domain is the core structural domain that mediates dimer formation, and dimer formation is used as a building block for the formation of large dynamic oligomers (56). Small molecule HSP lacks enzymatic activity and acts only as a retention enzyme, interacting with other cellular chaperone proteins to bind and protect denatured or unnatural proteins from aggregation (96). The BAG cochaperone 3 (BAG3) binds HSP20, HSP27 and HSP22 (HSPB 8), bridges both HSP22 and HSP70 and modulates both families to refold denatured luciferase, so that BAG 3 serves as an important co-chaperone protein (112, 113).

## 3 Heat shock protein and DPN

During the pathological process of DPN, HSP family members exhibit characteristic expression patterns and dynamic changes, which together participate in building a complex neuroprotective network. Through in-depth study of the mechanism of HSP action on nerves and HSP-based targeted therapeutic regimens, we can explore the potential therapeutic strategies of HSP in DPN (**Table 1**).

**Table 1:** Role of HSP in diabetes, DPN and other neuropathies.

Reference	Protein	Model	Controlmechanism	Conclusions
(117)	HSP90/KU-32	Animal model: STZ-induced HSP70 KO mice	HSP90 CTD inhibitor (KU-32) inhibits neuromodulatory protein-induced demyelination of myelinated SC and sensory neuron co-culture systems in an HSP70-dependent manner.	Studies have shown that targeting the HSP90 molecular chaperone can reverse the sensory and nociceptive hyperalgesia associated with DPN.
(80)	HSP90	Cell model: Yeast strains JY002 (tom70::HIS3),JY009 (tom70::HIS3 tom20::TRP1 p416ADH/TOM20 [URA3]) and Cos7 cells	HSP90 and HSP70 rely on the HSP90 ATPase binding to the TPR domain of the outer mitochondrial membrane receptor Tom70.Ensuring that precursor proteins are delivered precisely and efficiently to the mitochondria to perform their series of functions.	Chaperones of the HSP70 and HSP90 classes bind to the precursors(ADP/ATP carrier) upon their synthesis on cytosolic ribosomes. The chaperones not only guide the precursors to mitochondria but also specifically bind to the receptor Tom70 and thus ensure a direct transfer of the precursors to the import machinery of mitochondria.
(119)	HSP90/KU-596	Animal model: HSP70.1/70.3 double knockout (HSP 70 KO) mice	KU-596 disrupts the HSP90-HSF1 complex, induces HSP70 expression, improves mtBE, enhances MnSOD activity, and reduces mitochondrial superoxide levels in diabetic neurons.HSP70 translocates to depolarised mitochondria, and recruits parkin proteins to initiate clearance of damaged mitochondria.	KU-596 improves DPN through a multi-target mechanism (inhibition of oxidative stress, enhancement of mitochondrial function, and modulation of autophagy) and is superior to single-pathway inhibitors.
(123)	HSP90/DMAG	Animal model: STZ-induced diabetic apolipoprotein E-deficient mice	DMAG upregulated HSP70 expression, inhibited the NF-kB/STAT signalling pathway to reduce macrophage and T-cell infiltration, and inhibited the expression of factors such as TNF-α and TGF-β.	HSP90 is a key hub for diabetic complications, and its inhibitors ameliorate diabetic complications by inducing HSP70 and inhibiting the NF-kB/STAT pathway.
(124)	HSP90/AUY922	Animal model: db/db mouse	AUY922 significantly inhibits JNK1 phosphorylation in cells, protects cells and improves insulin signalling.	HSP90 inhibitors reversed hyperglycaemia in diabetic db/db mice and improved insulin sensitivity in insulin-resistant obese mice, further supporting the concept that the HSF1 pathway is a potentially viable anti-diabetic target.
(127)	HSP90/KU-177	Animal model: rTg4510 mice Cell model: iHEK-P301L cells and luciferase expressing PC3-MM2 cells	KU-177 disrupts the ability of the interaction between HSP90 and Aha1, blocks Aha1-driven HSP90-mediated Tau aggregation.	KU-177 can block Aha1-driven HSP90-mediated Tau aggregation., and it does not interfere with the normal folding function of HSP90.It can avoid the toxicity of global client protein degradation. A new strategy for the treatment of peripheral and central neuropathy.
(128)	HSP70	Molecule model:Protein Data Bank (PDB) code: 3HSC, PDB code: 1DKZ, PDB code: 4B9Q, PDB code: 1QVR	In the ADP-bound or nucleotide-free state, the nucleotide-binding domain of HSP70 connects to the substrate-binding domain, trapping peptide substrates within the binding cavity. Upon ATP binding, HSP70 undergoes a conformational change to form a specific binding site. This facilitates rapid exchange of peptide substrates. Subsequently, ATP is hydrolysed, and the substrate is encapsulated within the binding cavity.	HSP70 binds extended fragments of unfolded polypeptide chains to prevent misfolding and aggregation, maintains the substrate in an unfolded state, facilitates its spontaneous folding once it reaches the correct cellular localisation, and collaborates with HSP100 to depolymerise large protein aggregates.
(129)	HSP70	Animal model: B6SJL-TgN(SODI -G93A)1Gur^dl^ mices Cell model: Neuro2a cells	HSP70 preferentially binds to mSOD1 monomers, whilst CHIP interacts with HSP70 via its TPR and U-box domains. Subsequent ubiquitination of HSP70 bound to SOD1 facilitates the degradation of mSOD1.	HSP70 interacts with CHIP to promote the degradation of misfolded proteins recognised by HSP70 (e.g. mSOD1) to maintain intracellular homeostasis.
(130)	HSP70	Animal model: STZ-induced diabetic mice with HSP 70 KO	HSP70 mediates the downregulation of NOX2 expression.	HSP70 leads to a reduction in ROS production and ameliorates oxidative stress and inflammatory damage in sensory neurons in the diabetic state.
(131)	HSP70	Animal model: spontaneously hypertensive rat (SHR)	HSP70 interacts with caveolin-1 on the membrane of renal proximal tubule cells and is involved in signalling after angiotensin II type 1 receptor (AT_1_R) endocytosis. Membrane translocated HSP70 binds to the NOX4 subunit and down-regulates its expression, inhibiting NOX activity and reducing ROS production.	HSP70 is not limited to molecular chaperone roles, but exerts dynamic regulatory functions in response to oxidative stress through subcellular translocation (cytoplasm → plasma membrane) and targeted interactions (caveolin-1/NOX4).
(132)	HSP70	Animal model: spontaneously hypertensive rat (SHR)	HSP70 inhibits NOX activity and reduces ROS generation by directly binding to the NOX4/p22phox complex.	HSP70 modulates oxidative stress and cytoskeletal signalling, providing a novel intervention strategy for ROS-mediated injury.
(135)	HSP70	Animal model: 2CLP-induced sepsis in SD rats Cell model: mouse lung epithelial 12 cells	Enhanced HSP70 expression stabilises IkBα and impairs the activity of the intracellular inflammatory activator NF-kB.	The mechanism by which HSP70 affects NF-kB and other multimeric proteins may be of value in studies to treat other inflammation-related diseases.
(136)	HSP70	Cell model: HSP70-, HSP70C- and IκBαDN-inducible HeLa cells, Cos-1 cells were transfected with Gal4-Luc	HSP70 specifically binds IKKγ, which in turn inhibits IKK activity, which in turn inhibits NF-kB-mediated induction of anti-apoptotic genes and promotes tumour necrosis factor-mediated apoptosis.	Tumour necrosis factor-triggered apoptosis is initiated or exacerbated when HSP70 expression is increased to high levels that can disrupt NF-kB signalling.
(137)	HSP70	Animal model: adenovirus injection followed by lipopolysaccharide (LPS)-induced rat	Overexpression of HSP70, which inhibits LPS-induced nuclear translocation of NF-kB p65 and degradation of IkBα, prevents LPS-induced elevation of TNF-α and IL-6 levels in rats.	HSP70 may regulate cytokine expression in animals by inhibiting NF-kB, which contributes to the understanding of the regulatory role of HSP70 in inflammation and infectious diseases.
(138)	HSP72	Animal model: High-fat diet (HFD)-fed HSP72 transgenic mice (HSP72+/+)	HSP72 synergises with CHIP to inhibit JNK activation. In addition, HSP72 promotes the translocation of nuclear-encoded proteins to mitochondria via Tom70 receptor, maintains mitochondrial respiratory function and reduces ROS generation.	HSP72 demonstrated significant metabolic protection against obesity-induced insulin resistance by inhibiting JNK inflammatory signalling and enhancing mitochondrial oxidative capacity, providing a new target for the treatment of type 2 diabetes and related complications.
(125)	HSP72	Animal model: HFD-fed HSP 72 Tg mice	HSP72 activates AMPK/SIRT1, upregulates mitochondrial transcription factor A (Tfam), increases mitochondrial number and enhances oxidative metabolism.	HSP72 provides a new strategy for metabolic diseases and related mitochondrial dysfunction by promoting mitochondrial generation.
(139)	HSP70	Animal model: MPZ-Raf+/+×HSP 70 KO mouse	KU-596 reduces c-Jun expression in SC and ameliorates demyelinating neuropathy by inducing HSP70 and enhancing proteasomal degradation of c-Jun.	HSP70, by targeting c-Jun clearance, may be a new hub for intervention in demyelinating lesions.
(140)	HSP70	Cell model: human monocyte	Exogenous HSP70 interacts with the CD14 receptor, leading to the phosphorylation of IkBα, which in turn activates NF-kB, thereby inducing elevations of TNF-α, IL-1β, and IL-6.HSP70 can also be dependent on an unknown receptor, selectively inducing TNF-α via calcium inward flow.	In addition to its function as an intracellular molecular chaperone, HSP 70 acts as a potent cytokine in the extracellular milieu, influencing the functional properties of immunoreactive cells. This dual role as chaperone and cytokine helps to provide new strategies for future research and clinics.
(141)	HSP70	Cell model: human monocyte	Exogenous HSP70 mediates inflammatory factor expression through the MyD88/IRAK/NF-kB signalling pathway. TLR 2/4 was also used to mediate pro-inflammatory signalling in a CD14-dependent manner.	The mechanism of action and biological function of exogenous HSP70 were elucidated, which is sufficiently paved for the development of efficient pharmacological or molecular tools.
(142)	HSP70	Animal model: retinal ischaemia-reperfusion (IR) injury in TlR4-, Myd88- and Trif-KO mice	eHSP70 acts as a damage-associated molecular pattern (DAMP), and PKC acts as a molecular switch in the HSP70/TLR4 signalling cascade, the activation of which amplifies the pro-inflammatory response and mediates neuronal apoptosis. eHSP70 preferentially up-regulates Myd88 in astrocytes, which mediates TNF-α and promotes neuronal apoptosis through the TLR4/Myd88 signalling cascade.	HSP70 can mediate TLR4-dependent innate immune responses only when PKC is activated, a mechanism that can provide new insights into the capacity of other DAMP-mediated immune responses.
(143)	HSP70	Genetically engineered human cell models, murine cell models for experimental needs	HSP70-peptide complex (HSP70-PC), after uptake by antigen-presenting cells (APCs), cross-presents antigens via the antigen-processing (TAP)-dependent MHC-I pathway and activates intrinsic immunity and induces pro-inflammatory factors (IL-1β, TNF-α, IL-6) through the TLR2/4-CD14-MyD88/IRAK/NF-kB signalling pathway. In this process, the scavenger receptor LOX-1 binds HSP 70 with high affinity.	LOX-1 is a key high-affinity receptor for HSP70. These findings expand mechanistic knowledge of HSP70 in immune surveillance, stress response, and chronic diseases, and provide a theoretical basis for therapeutic strategies targeting the HSP70 receptor.
(144)	HSP70	Cell model: male athlete blood cells	eHSP70 stimulates the secretion of pro-inflammatory factors such as TNF-α, IL-1β, and IL-6 by binding to CD14. eHSP70 is also known for its ability to directly activate the complement cascade. Its binding to CD94 on the surface of NK cells (which forms a complex with NKG2 chains) enhances NK cell proliferation, cytotoxicity, and killing. eHSP70 also directly activates the complement cascade response, amplifying the inflammatory response and protecting many organ systems from irreversible cellular damage.	iHSP70 functions as a guardian of protein homeostasis, whereas eHSP70 is a key regulator of intrinsic immunity. Exercise stress orchestrates the acute inflammation-anti-inflammation balance by elevating eHSP70 and provides a molecular basis for exercise immune intervention.
(145)	HSP70	Animal model: B6.129S7-HSPa 1a/HSPa 1btm mice	eHSP70 acts as a DAMP, activating neutrophils and macrophages and activating the complement system by binding to immune cell receptors (e.g. TLR2/4). eHSP70 deletion resulted in virtually no expression on neutrophils and macrophages in a mouse model of muscle injury, and exogenous injection of recombinant HSP70 (rHSP70) restored its immune cell infiltration.	eHSP70 acts as a DAMP to activate immune cells and initiate inflammatory clearance.
(146)	HSP70	Obese T2DM and non-obese T2DM male patients	Obesity or T2DM leads to increased secretion of the pro-inflammatory factor TNF-α, thereby activating kinases such as JNK and IKK. Subsequent inhibition of HSF1 results in downregulation of iHSP72, exacerbating insulin resistance and disrupting protein homeostasis. Hyperglycaemia or oxidative stress prompts immune cells to release eHSP72, thereby intensifying inflammation.	iHSP72 expression in skeletal muscle of T2DM patients was positively correlated with insulin sensitivity, and adiposity was negatively correlated with iHSP72 expression. Serum eHSP72 was positively correlated with disease duration and positively correlated with inflammatory markers (CRP, TNF-α). In T2DM, the imbalance between iHSP72 down-regulation and eHSP72 up-regulation reflects the pathological role of obesity and inflammation, and targeting the HSP70 pathway may provide a new strategy for the treatment of diabetes and its complications.
(147)	HSP72	27 females and 23 males: 63.4 ± 4.4 years of age, BMI (body mass index)=25.5 ± 2.7 kg/m^2^	eHSP72 binds to TLR2/4, activating the JNK and IKK inflammatory pathway. This leads to mitochondrial dysfunction, increased metabolic stress in β-cells, and reduced insulin secretion. Consequently, β-cell viability diminishes, culminating in progressive exhaustion.	Plasma eHSP72 levels were positively correlated with insulin resistance index, TNF-α, cortisol, and leptin/lipocalin. It is suggested that chronic exposure to eHSP72 may mediate β-cell failure.
(125)	HSP72/BGP-15	Animal model: mice overexpressing HSP72 in skeletal muscle (HSP72Tg)	Overexpression of HSP72 upregulates Tfam, increases mitochondrial number by 50% and inhibits JNK activation.	BGP-15 increases mitochondrial number and insulin sensitivity in a rat model of T2DM. The increased oxidative metabolism associated with HSP72 activation has potential clinical significance not only for T2DM but also for other diseases with impaired mitochondrial function.
(149)	HSP27	Cell model: murine L929	HSP27 upregulates enzymes in the ROS-glutathione pathway, such as glucose-6-phosphate dehydrogenase, glutathione reductase, and glutathione transferases. HSP27 assists in maintaining glutathione in its reduced state, thereby enhancing its capacity to scavenge reactive oxygen species.	HSP27 upregulates GSH levels, thereby enhancing antioxidant capacity.
(150)	HSP27	Cell model: mouse cell line expressing HSP27	High-level expression of HSP27 down-regulates cytochrome C release from mitochondria while regulating F-actin polymerisation and inhibiting the mitochondrial apoptotic pathway.	HSP27 can act as a fibre stabilising factor under stress and intervene in cytoskeletal dynamics by regulating actin polymerisation, which in turn inhibits the activation of apoptotic signalling pathways.
(152)	HSP27	Isolated human retina Animal models: retina of genetically engineered mice defective in TNF-α receptor-1 (P-55 knockout), retina of genetically engineered mice defective in TNF-α receptor-2 (P-75 knockout)	Exogenous HSP27 antibody enters human retinal neuronal cells through an endocytosis mechanism, and after internalisation, HSP27 antibody activates a protein hydrolysis cascade, which involves caspase-8 and caspase-3 activation as well as PARP cleavage, which in turn induces apoptosis.	Abnormal exposure of HSP27 under stress conditions may be recognised by the immune system and then induce the production of auto HSP27 antibodies, leading to the destabilisation of the cytoskeleton, inducing apoptosis of neuronal cells and increasing the risk of nerve injury.
(153)	HSP25	Animal models: STZ-induced diabetic rat glomeruli, isolated rat glomeruli exposed to free radical stress (H2O2)	Highly expressed HSP25 is phosphorylated in a p38MAPK-dependent manner, altering its oligomeric state and stabilising the actin cytoskeleton, leading to loss of contractile function in tethered cells.	HSP25/HSP27 are expressed at high levels in target organs of diabetic complications, revealing their possible involvement in the response mechanism to chronic injury in diabetes.
(154)	HSP27	Animal models: pre-diabetic BB/Wor rats, age-matched non-diabetic susceptible BB rats	The expression of caspase-3 and pro-apoptotic protein Bax was up-regulated in DRG and induced the expression of anti-apoptotic Bcl-Xl as well as HSP27 and HSP70.	Bcl-Xl as well as HSP27 and HSP70 prevent apoptotic stress in diabetic DRG.
(155)	HSP27	Animal model: STZ-induced diabetic rat	Elevated HSP27 expression in trophin-deprived diabetic neurones may represent an attempt to mitigate this state and maximize survival.	Elevated mRNAs other than HSP27 were not observed after damage to diabetic DRG sensory neurons.
(157)	HSP27	T2DM patients with normal cognitive function and T2MD-MCI patients	Oxidative stress and ferroptosis are both closely associated with T2DM-MCI. HSP27 leads to a reduction in ROS levels. HSP27 regulates iron homeostasis, thereby preventing ferroptosis caused by the excessive accumulation of intracellular iron.	In T2DM-MCI patients, plasma HSP27 levels were decreased, and the risk of MCI development was significantly increased when HSP27 levels were below 3.51 pg/mL (OR = 0.355), with a specificity as high as 94.4%, suggesting that HSP27 is expected to be a biomarker for diabetic nerve-related lesions.
(158)	HSP25	Animal model: HSF1 KO mice	HSF1 deficiency reduces HSP25 expression, and downregulation of HSP25 is consistent with a significant reduction in the glutathione (GSH)/oxidised glutathione (GSSG) ratio and reduced glucose 6-phosphate dehydrogenase (G6PD) activity.	Down-regulation of HSP25 expression resulted in increased oxidative stress and ROS generation. Upregulation of HSP25 expression attenuated oxidative stress and ROS generation.
(163)	HSP90/DDO-6691	Cell models: CDC37 knockout cells, HCT-116 cells	The PPI between HSP90 and CDC37 plays a positive role in tumourigenesis, and the small molecule inhibitor DDO-6691 targets HSP90 by covalently binding to it, interfering with complexation with CDC37, and targeting the disruption of the HSP90-CDC37 interaction.	The findings highlight the therapeutic promise of DDO-6691 for targeting covalent HSP90-CDC37 complexes.
(165)	HSP70/JDM37	Cell model: HEK 293 T cells	JDM37 significantly promotes HSC70 ATP hydrolysis, which recruits HSP70 to specific client protein complexes and regulates pathogenic protein condensates such as RIα and EML4-Alk.	In this study, JDM37, the first functional artificial HSP70 activator, was created by computational protein design to break through the limitations of natural JDP, and its modularity provides a new tool for targeting protein-phase-separation-related diseases (e.g. neurodegenerative diseases, cancers), as well as opening a new pathway for elucidating the molecular mechanism of HSP70-JDP.
(168)	HSP27/TAT-HSP27_65−90_	SAH patients, normal pressure hydrocephalus (NPH) patients Animal model: rat SAH model	Overexpression of HSP27 effectively inhibited the activation of MKK4, JNK, c-Jun and caspase-3 elevated by SAH.TAT-HSP27_65–90_ effectively inhibited hemolysate-induced neuronal apoptosis.	TAT-HSP27_65–90_ ameliorates neurological deficits by reducing apoptosis.

### 3.1 Expression characteristics and regulatory mechanisms of major HSP families in DPN

#### 3.1.1 Structural characterisation of HSP90 and the mechanism of DPN regulation by its inhibitors

**Figure 3:**
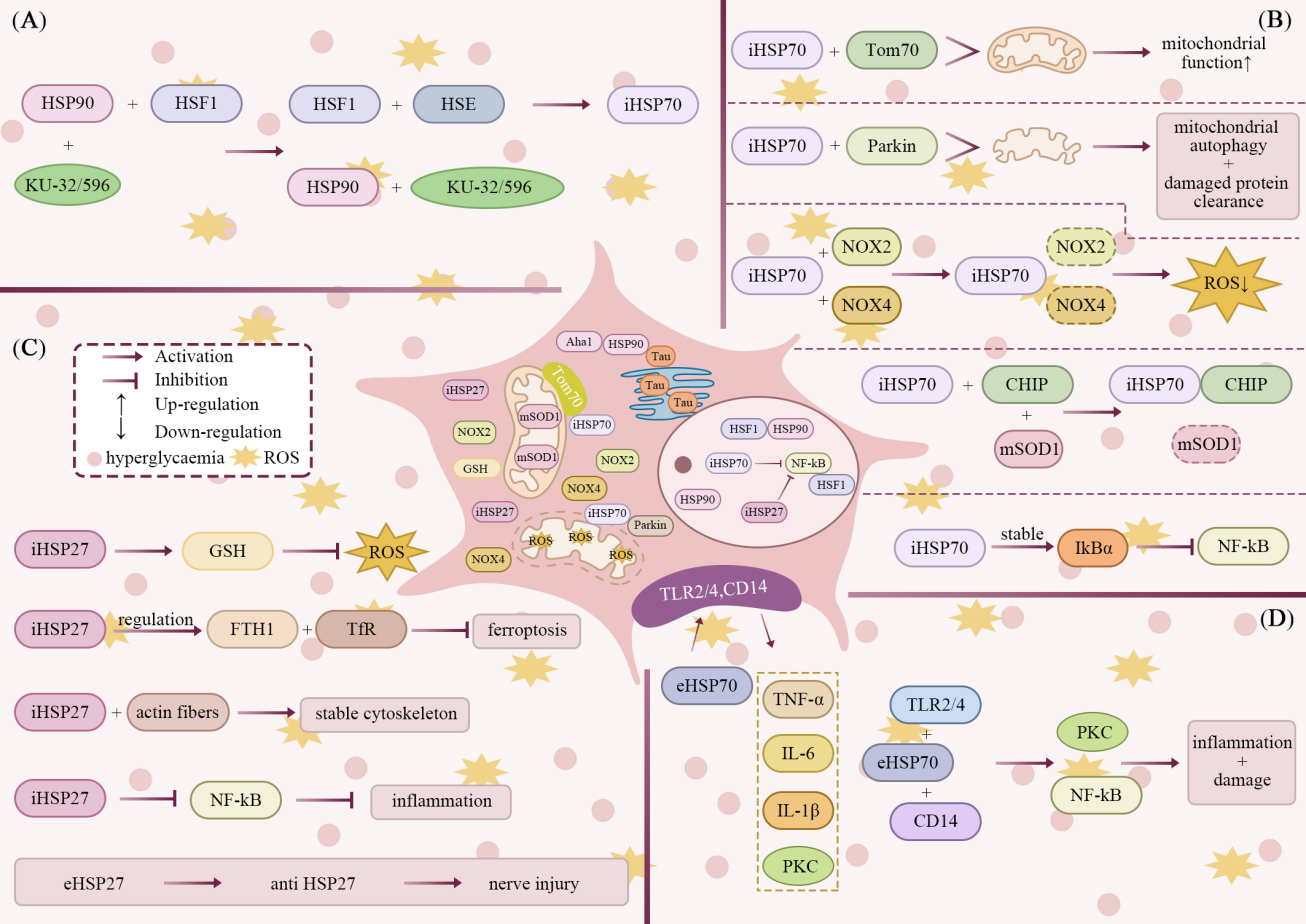
Mechanisms of action of HSP on neuronal cells under hyperglycaemia and stress. **(A)** HSP90 CTD inhibitors (KU-32/596) promote the activation of HSF1. Activated HSF1 binds to the Heat Shock Element (HSE) and induces the upregulation of iHSP70 expression. **(B)** iHSP70 mediates the transport of nuclear-encoded proteins to mitochondria by binding to Tom70. This enhances mtBE and improves mitochondrial function. iHSP70 translocates to depolarised mitochondria and recruits Parkin, thereby promoting mitochondrial autophagy and the clearance of damaged proteins. iHSP70 regulates NOX2/4 expression, downregulating their levels to diminish oxidative stress. CHIP promotes the ubiquitination and degradation of misfolded proteins (such as mSOD1) recognised by iHSP70, thereby maintaining intracellular homeostasis. Regarding inflammatory regulation, iHSP70 inhibits NF-kB activation by stabilising IkBα, consequently reducing inflammatory responses. **(C)** iHSP27 can upregulate GSH levels, thereby reducing oxidative stress. iHSP27 inhibits ferroptosis by regulating key proteins in iron homeostasis: ferritin heavy chain 1 (FTH1) and transferrin receptor protein (TfR). Furthermore, iHSP27 regulates actin to stabilise the cytoskeleton. Acting as an inflammatory modulator, it inhibits the NF-kB signalling pathway, thereby reducing inflammatory responses. However, eHSP27 may induce anti HSP27 antibodies, potentially increasing the risk of neurological damage. **(D)** eHSP70 can upregulate multiple inflammatory cytokines via the CD14-dependent TLR2/4 pathway, activating the PKC pathway and NF-kB. This consequently triggers inflammatory responses and neural damage.

(**Figure 3**) As mentioned earlier, HSP90, as a molecular chaperone protein, forms a functional homodimer through its CTD and relies on NTD ATPase activity to direct protein folding into a biologically active conformation under the synergistic effect of co-chaperone proteins. In unstressed cells HSP90 constitutively binds and represses heat shock factor 1 (HSF1). Proteotoxic stress or small-molecule ligands that alter HSP90 conformation disrupt this interaction, thereby derepressing HSF1 and driving transcription of cytoprotective genes including HSP70 (114). HSP90 CTD contains an ATP-binding structural domain that weakly binds the antibiotic neomycin (115). KU-32 is an HSP90 CTD-specific inhibitor optimised on the structure of the coumarin ring pharmacophore of neomycin, which modulates the function of HSP90 by mutation and induces the heat shock response to up-regulate the expression of HSP70 (116). KU-32 protected unmyelinated embryonic sensory neurons from glucose-induced cell death in an HSP70-dependent manner and ameliorated neuromodulatory protein-induced demyelination of myelinated SC and sensory neuron co-culture systems. However, in HSP70 knockout diabetic mice, KU-32 completely lost its therapeutic effect, suggesting that its neuroprotective effect is dependent on the presence of HSP70 (117). This mechanism of action may be related to the fact that HSP70 mediates the transport of nuclear-encoded proteins to mitochondria and improves the function of the respiratory chain (80, 118).

Similarly, KU-596 is a conformational modulator that binds to HSP90 CTD to induce a conformational change and affects the interaction of NTD and co-chaperone proteins to derepress HSF1 and induce HSP70 expression. *In vitro* experiments showed that KU-596 was effective in reducing superoxide levels and improving mitochondrial bioenergetics (mtBE) only in neurons of HSP70 wild-type diabetic mice, but had no significant effect in neurons of HSP70 knockout mice. The effect of KU-596 in improving mtBE was dependent on HSP70 in high glucose environment, but part of its effect could be independent of HSP70 in normal glucose conditions. In addition, although KU-596 reduced mitochondrial superoxide levels, this effect was not dependent on manganese superoxide dismutase (MnSOD). However, the ameliorative effect of KU-596 on maximum respiratory capacity (MRC) of diabetic neurons was lost when MnSOD expression was down-regulated, suggesting that its mechanism of improving mtBE is not directly causally related to superoxide scavenging (119). HSP70 is a key factor in Parkin-mediated mitochondrial autophagy: it translocates to depolarised mitochondria and recruits Parkin, thereby facilitating clearance of damaged proteins (120). In contrast, diabetic neurons often show significant mitochondrial depolarisation, suggesting that the neuroprotective mechanism of KU-596 may be closely related to the HSP70-dependent mitochondrial autophagy pathway (119, 121, 122).

In addition, geldanamycin derivatives (DMAG) and AUY922 act as HSP90 NTD inhibitors, binding to the N-terminal nucleotide-binding pocket of HSP90, blocking the folding of client proteins and promoting their degradation. DMAG has been shown to ameliorate diabetic nephropathy by inhibiting the activation of the NF-kB and STAT signalling pathways and by decreasing the expression of inflammatory genes. But it does not affect blood glucose or glycated haemoglobin levels (123). In contrast, AUY922 more effectively regulates the HSP90 complex in skeletal muscle, adipose tissue and liver of diabetic patients (124). BGP-15, a nicotinic kainamide oxime derivative, directly activates HSF1 and promotes HSP70 expression, thereby improving insulin resistance and mtBE (125).

Among the HSP-regulated client proteins, the microtubule-associated protein Tau (MAPT, Tau) is the most representative, and its oligomeric aggregation is a key driver in the development of neurodegenerative diseases (i.e. Tauopathy). Aha1 is the only known co-molecular chaperone that enhances the activity of the HSP90 ATPase, which synergistically induces the formation of Tau protofibrils in conjunction with HSP90. KU-177, an inhibitor of Aha1-HSP90 interaction, can block the process of Tau aggregation, thereby alleviating the neurodegenerative pathological injury (126, 127).

#### 3.1.2 Dual role of HSP70 in DPN and regulatory mechanisms

HSP70 (including isoforms such as HSP72 and HSPA1A) is one of the most intensively studied stress proteins. Notably, there are significant functional differences between intracellular HSP70 (iHSP70) and extracellular HSP70 (eHSP70). iHSP70 serves multiple critical functions in protein homeostasis. It facilitates the folding of nascent peptides, corrects misfolded proteins, and manages protein aggregates. Additionally, iHSP70 mediates the degradation of dysfunctional proteins. These activities collectively enable iHSP70 to play a vital protective role in terminally differentiated cells such as neurons. One key neuroprotective mechanism is the clearance of protein aggregates implicated in DPN (128). CHIP promotes ubiquitination and degradation of misfolded proteins recognised by iHSP70 (e.g. Mutant superoxide dismutase 1,mSOD1) to maintain intracellular homeostasis (129). In addition, iHSP70 negatively regulates the expression of NADPH oxidase 2 (NOX2) and attenuates oxidative stress damage in sensory neurons in diabetic state (130). In vascular smooth muscle cells, immunoprecipitation and knockdown experiments also confirmed the involvement of iHSP70 in the regulation of NOX4 (NADPH oxidase 4) degradation (131, 132). It has been reported that only about 1% of the proteins in mitochondria are encoded by mitochondrial DNA, and the remaining about 99% of the proteins need to be imported into mitochondria after nuclear transcription and cytoplasmic translation (118, 133). iHSP70 mediates the translocation of nuclear-encoded proteins to mitochondria by binding to the mitochondrial outer membrane receptor Tom70 (80). In mitochondria, HSP70 and the endosomal receptor Tim44 work together to form an ATP-dependent molecular “motor” that propels precursor proteins into the matrix region (134). Notably, mitochondrial dysfunction is closely associated with the development of DPN. In terms of inflammatory regulation, iHSP70 inhibits NF-kB activation by stabilising the protein IkBα (NF-kB inhibitory protein α), restricting phosphorylated IkBα proteasomal degradation, and binding to IKK (IkB kinase) complexes (135, 136). However, TNF-triggered apoptosis is initiated when iHSP70 expression is increased to high levels to disrupt NF-kB signalling (136). A study indicates that iHSP70 overexpression inhibits adenoviral post-injection lipopolysaccharide (LPS)-induced elevation of IL-6 and TNF-α levels in the blood of rats, as well as inhibiting the expression of IL-6 and neutrophil chemokine-1 (135, 137). In addition, iHSP72 inhibits the phosphorylation of c-Jun (a transcriptional regulator, a member of the leucine zip family) N-terminal kinase (JNK), thereby attenuating high-fat diet-induced insulin resistance (125, 138). Mechanistic studies further show that HSP70 preserves myelin structure by blocking c-Jun driven SC dedifferentiation and subsequent demyelination (139).

In contrast, eHSP70 may induce a pro-inflammatory response through the LPS receptor (CD14)-dependent TLR2/4 (Toll-like receptor 2/4, Gram-positive/negative bacterial receptor) pathway, activating the expression of a variety of inflammatory factors, such as IL-1β, IL-6 and TNF-α (140–142). Meanwhile, PKC is a molecular switch in the HSP70/TLR4 signalling cascade and activation of PKC amplifies the pro-inflammatory response and mediates neurocyte apoptosis (142). eHSP70 functions as an endogenous danger signal that activates innate immunity. This immune activation is characterized by the upregulation of major histocompatibility complex II (MHC-II) and CD86 on antigen-presenting cells, along with the production of pro-inflammatory cytokines including IL-1β, TNF-α, and IL-6. These effects are mediated through the TLR2/4-CD14-MyD88/IRAK/NF-kB signaling axis and downstream antigen-processing pathways (143). In addition, eHSP70 acts as a chemokine, recruiting macrophages, neutrophils and natural killer (NK) cells (144, 145). Of interest, eHSP70 levels are positively correlated with the degree of systemic inflammation in patients with type 2 diabetes mellitus (146). It also induces pancreatic β-cell dysfunction and even apoptosis (147). Therefore, HSP70 exhibits the dual characteristics of “protector and blade” in DPN, and its cellular localisation determines its differential impact on disease progression.

#### 3.1.3 Neuroprotective effects and biomarker value of HSP27

HSP27 (also known as HSPB1) is an important member of the family of small-molecule heat shock proteins, a highly conserved peptide protein closely related to actin with a molecular weight of approximately 27 kDa (148). In the pathological process of DPN, HSP27 has a dual biological regulatory function, which not only plays a role in neuroprotection, but may also be involved in pathological injury mechanisms. It plays a key role in cytoprotection and migration, in addition to possessing molecular chaperone activity. HSP27 expression can increase cellular glutathione (GSH) levels, as observed in unstressed mouse cells. However, this effect is context-dependent and is not always evident in cells with constitutively high HSP27 expression, such as HeLa cells. Supporting this, fibroblasts expressing HSP27 show transcriptional up-regulation of ROS-GSH pathway enzymes. This pattern highlights the cytoprotective and migratory functions of HSP27 (149). HSP27 also acts as a fibre stabilising factor under stress and intervenes in cytoskeletal dynamics by regulating actin polymerisation, thereby inhibiting the activation of apoptotic signalling pathways (150). Studies have shown that intracellular HSP27 is an important regulator of neuronal survival and axonal regeneration (151). However, its overexpression under stress conditions and aberrant extracellular exposure induces an immune response and the production of anti HSP27 autoantibodies, which mediate neuronal apoptosis and increase the risk of nerve injury (152).

Diabetes-related studies have revealed that the expression of small molecule heat shock proteins (including HSP25 and HSP27) is significantly upregulated in target organs related to diabetes complications such as glomerulus, dorsal root ganglion (DRG) and retina, suggesting that they may be involved in the mechanism of response to chronic diabetes injury (153–156). 2025 A study in patients with type 2 diabetes mellitus with mild cognitive impairment (T2DM-MCI) showed a significant decrease in plasma HSP27 levels and a significant negative correlation with the speed of information processing (Trail Making Test Part A, also known as TMTA) and executive function (Trail Making Test Part B, also known as TMTB). Multiple linear regression analysis further showed that the risk of developing MCI was significantly increased (OR = 0.355) when the HSP27 level was below 3.51 pg/mL, with a specificity as high as 94.4%, suggesting that HSP27 is expected to be a sensitive biomarker for the identification of diabetes-related neurological dysfunction (157).

In the exploration of molecular mechanisms, HSP27 exerts multiple neuroprotective effects mainly through its conserved α-crystalline structural domain. On the one hand, HSP27 effectively scavenges ROS and exerts antioxidant protection in the diabetic microenvironment, thereby alleviating the neurological damage induced by oxidative stress (158–160). On the other hand, in diabetic hyperglycaemic states, HSP27 also inhibits the ferroptosis process by regulating key proteins of iron homeostasis (161). In addition, HSP27 can also act as an inflammatory regulator and reduce the expression of inflammatory factors such as TNF-α and IL-6 by negatively regulating the NF-kB signalling pathway, thereby improving the inflammatory microenvironment of neural tissues (162).

### 3.2 Strategies and recent advances in targeting HSP therapy

#### 3.2.1 HSP90: a new perspective from tumour targets to DPN therapy

The regulatory role of HSP90 inhibitors in DPN has been elaborated above. Notably, in recent years, targeting the HSP90 molecular chaperone system has gradually become a highly promising strategy for tumour therapy. Studies have shown that the protein-protein interaction (PPI) between HSP90 and CDC37 plays a key role in the process of tumourigenesis. Disrupting this PPI, the maturation of oncogene kinase-like client proteins can be specifically inhibited, which opens up new avenues for cancer therapy. Compared with traditional HSP90 ATPase inhibitors, HSP90-CDC37 PPI inhibitors have higher target specificity and are expected to reduce the occurrence of non-specific side effects such as heat shock reaction (HSR). The small molecule inhibitor DDO-6691 targets HSP90 via covalent binding and disrupts its complex with CDC37. This finding demonstrates the therapeutic potential of directly targeting the HSP90-CDC37 interaction. Furthermore, it provides a new mechanistic avenue for the development of cancer therapeutics (163). In addition, its unique covalent binding mechanism provides a new idea for the development of DPN-targeted therapeutic drugs.

#### 3.2.2 HSP70: a cutting-edge target for protein homeostasis regulation

The AGEs pathway is closely related to DPN. Studies have shown that hot tub therapy (HTT) can alleviate the complications associated with diabetic rats. This mechanism may involve an increase in extracellular HSP70 levels, which can help to protect the protein structure and prevent the formation of AGEs (164). However, the aforementioned HTT study had several limitations. It did not assess HSP70 expression levels or their interactions with neuroprotective factors in neural tissues. Furthermore, it lacked functional and structural assessments, such as nerve conduction velocity measurements and histopathological evaluation of myelin integrity. These aspects warrant verification in subsequent investigations. This non-invasive physiotherapy may be a safe and cost-effective intervention for DPN. HSP70 and its co-chaperone JDP are core mediators of the protein quality control (PQC) system, jointly regulating the folding and degradation of client proteins. Imbalance of PQC function leads to the accumulation of aberrant proteins, which can lead to a wide range of disorders. The main function of JDP, also known as HSP40s, is to recruit HSP70 to specific client protein complexes (165).The typical human JDP protein DnaJA2 consists of an N-terminal J-structural domain (JD), a glycine/phenylalanine (G/F) enriched region, two C-terminal structural domains (CTDI and CTDII), and a dimerisation motif.

Zhang et al, designed a class of proteins called J-domain mimics (JDM) that are able to bind HSP70 at the same sites as natural JDs. Of the multiple JDMs designed, most inhibit ATP turnover by HSP70, while JDM37 promotes ATP hydrolysis with an efficacy comparable to that of natural DnaJA2, and shows a high affinity for HSP70 (165). The study used a complex (JDM37-GFPnb-mCh) that fused JDM37 with a green fluorescent protein (GFP) nano antibody (GFPnb) and a red fluorescent protein (mCherry) in order to track its localisation and disruption of GFP-containing cohesions, and it was shown that JDM37 did indeed dissolve intracellular cohesions by binding and activating HSP70 (165). Utilizing JDM37, a cohesion scrambler, the researchers investigated the role of intracellular cohesin RIα in signaling. They demonstrated that RIα segregates cyclic adenosine monophosphate (cAMP) and activates protein kinase A (PKA). This mechanism effectively spatially compartmentalizes PKA activity (165). Synchronisation of cAMP/PKA signalling, RIα and JDM37-mediated cohesion perturbation was achieved by mCherry labelling of RIα (RIα-mCh) and fusion of JDM37 with mCherry nano antibody (mChnb) and blue fluorescent protein (TSapphire) (JDM37-mChnb-TSapphire) visualisation (165). The results suggest that the recruitment of HSP70 to the cohesion by JDM37 is necessary for the perturbation process, and that this recruitment process may increase the probability of RIα being used as a substrate for HSP70, thereby modulating RIα cohesion. In addition, it was also shown that the oncogenic fusion protein EML4-Alk can be solubilised by the JDM37-HSP70 complex (165).

Abnormal protein aggregates such as Tau proteins and AGEs are widely present in the neural tissues of DPN and other degenerative neuropathies. The aforementioned HSP70 activators (e.g., JDM37) are able to disintegrate pathogenic protein aggregates similar to RIα and EML4-Alk, suggesting their potential therapeutic value in removing DPN protein aggregates. Drawing on Zhang et al.’s GFPnb/mChnb targeting strategy, combined with the JDM37 fusion technology, future development of nanobody-JDM37 fusion proteins targeting SC or neuron specificity is expected. Overall, the future research of DPN therapy could focus on the integrated strategy of “dissolving pathological protein aggregates” and “promoting the correct folding of neural repair proteins”.

#### 3.2.3 HSP27: a new strategy for targeting apoptosis and neuroprotection

HSP27 is an important member of the small heat shock protein family that reduces protein aggregation, promotes degradation of aberrant proteins, inhibits cytochrome-c release and activation of cysteinyl asparaginase (caspase) and achieves cytoprotection by stabilising the cytoskeleton and exerting an antioxidant effect (166, 167). In a rat model of subarachnoid haemorrhage (SAH), the level of HSP27 in cerebrospinal fluid (CSF) increased and then decreased significantly. Overexpression of HSP27 effectively alleviated SAH-induced neurological dysfunction and apoptosis in the basal cortex and inhibited the activation of mitogen-activated protein kinase kinase 4 (MKK4), JNK, c-Jun and caspase-3 (168). Since hyperglycaemia-induced oxidative stress and mitochondrial apoptosis play key roles in DPN, targeting this pathway is considered as a potential therapeutic strategy to slow down neurological damage. Lateral ventricle injection of Trans-activator of transduction-Heat shock protein 27 (amino acid residues 65–90) (TAT-HSP27_65-90_) has been found to exert neuroprotective effects, inhibit caspase-3-mediated mitochondrial apoptotic signalling and attenuate apoptosis in the basal cortex region of rats after SAH (168). Mitochondria are not only the centre of cellular energy metabolism, but also a key structure in the regulation of programmed cell death. Their dysfunction is closely related to a variety of metabolic diseases (e.g. hypertension, cancer and neurodegenerative diseases), posing a serious threat to human health (169, 170). However, there is a lack of drugs that can effectively target mitochondria (171). Cerrato, Kang et al. designed and developed a variety of novel mitochondria-targeted cell-penetrating peptides (CPP), such as mtCPP-1 and CAMP, which are able to modulate a wide range of biological processes within the mitochondria and enhance their biological effects (170, 172). Despite the potential of TAT-HSP27_65–90_ in DPN therapy, its efficacy in animal models (e.g. STZ-induced rats) still needs to be validated by further studies. In the future, its stability, mitochondrial targeting, and therapeutic specificity in DPN could be enhanced by structural optimisation.

## 4 Discussion

DPN is a common complication of diabetes mellitus, and its mechanism involves multiple pathway abnormalities such as oxidative stress, inflammation, and accumulation of AGEs triggered by hyperglycaemia (6–10). Currently, the treatment of DPN lacks a curative approach to the disease mechanism and relies mainly on symptomatic treatments with limited effects and obvious side effects. Existing therapies cannot reverse the disease, and some drugs (e.g., metformin) may even aggravate the disease (11, 12). Therefore, the treatment of the disease mechanism has become an important direction of exploration (17).

HSP is an important member involved in cell repair and regulation of protein homeostasis *in vivo*. It is the first line of defence against protein misfolding and accumulation of aggregation-prone proteins. It protects neuronal cells by inhibiting apoptosis, protecting the cytoskeleton and immunomodulation. Thus, HSP provides a potential target for the treatment of many neurological diseases (56).

This review elucidates the critical role of HSP in the pathogenesis and potential treatment of DPN. We systematically summarise the expression patterns, regulatory mechanisms and therapeutic targets of HSP90, HSP70 and HSP27. Highlighting their dual roles of neuroprotection and neurotoxicity in different cellular localisations and environmental stresses.

HSP90 is a key regulator of neuronal survival under hyperglycaemic stress. Its inhibitors (e.g., KU-32 and KU-596) exert neuroprotective effects primarily through induction of HSP70, which in turn enhances mitochondrial bioenergetics and reduces oxidative stress (117, 119). Notably, the efficacy of these compounds is lost in HSP70 knockout models, highlighting the dependence on HSP70-mediated pathways (117). In addition, HSP90 inhibitors (e.g. DMAG and AUY922) display anti-inflammatory properties by inhibiting NF-kB and STAT signalling, providing a multi-targeted approach to alleviate diabetic complications (123, 124). The recent development of covalent HSP90-CDC37 inhibitors (e.g. DDO-6691) is a promising strategy to achieve higher specificity and reduce off-target effects, potentially translating from oncology to DPN therapy (163).

HSP70 exemplifies the environmental duality of stress proteins. iHSP70 provides protection by promoting protein refolding, mitochondrial protein import via Tom70, and inhibition of inflammatory pathways such as NF-kB and JNK (80, 125, 130, 135, 138). Conversely, eHSP70 can act as a DAMP and activate TLR2/4-mediated pro-inflammatory responses leading to insulin resistance and β-cell dysfunction (140–142, 146, 147). This duality requires therapeutic strategies that enhance iHSP70 while minimising eHSP70 release. In addition, the novel HSP70 activator JDM37 disrupts pathological protein cohesions, providing a breakthrough approach to address the protein accumulation commonly seen in DPN (165).

HSP27 exhibits important neuroprotective effects mediated through its roles in antioxidant defence, apoptosis inhibition, and iron homeostasis (149, 150, 161). Its ability to inhibit caspase-3 activation and stabilise the cytoskeleton highlights its therapeutic potential (150, 168). However, extracellular exposure of HSP27 may cause autoantibody production, thereby exacerbating neuronal apoptosis (152). Clinically, reduced plasma HSP27 levels are strongly associated with T2DM-MCI, suggesting that it may serve as a sensitive biomarker for early detection of neurocognitive decline in diabetes (157).

This paper discusses HSP-centred therapeutic strategies, including physical interventions such as small molecule inhibitors, peptide therapies and hot tub therapies, representing a shift in DPN treatment from symptom management to targeting the pathomechanism (164). However, a number of challenges remain, and future therapies must enable cell-specific targeting (e.g. SC and neurons) to maximise efficacy and reduce systemic effects. Zhang et al. achieved precise localisation and disaggregation of intracellular GFP aggregates by fusing GFPnb with JDM37 (165). Myelin protein zero (MPZ) is specifically expressed in SCs (139). Based on this, we may infer that conjugating HSP70 activators (such as JDM37) or HSP70 inducers (such as KU-596) with antibodies or nanobodies that specifically bind to SC or neuronal surface markers (such as MPZ) could enable drug enrichment within diseased nerves, thereby reducing systemic exposure and off-target toxicity. Therefore, by drawing upon the selective induction mechanism of KU-596 and the high-affinity binding mode of JDM37, a new generation of “target-activate” dual-function molecules can be engineered to enhance therapeutic precision. The key to the pharmacological strategy for HSP is to distinguish its intracellular and extracellular functions, and to understand the definition conditions and secretion pathways of their role in the extracellular space. For example, inhibitors of intracellular HSP90, cytoplasm-delivered TAT-HSP27 peptides, and nanobody-mediated delivery of JDM37 each exploit precise functional sites of HSPs. Extracellular HSP90α(eHSP90α)is secreted by normal cells under stress and by tumour cells in response to oncogenic signals; nevertheless, the secretion pathway is still not fully defined. Once outside the cell, eHSP90α promotes processes such as cell migration and wound healing (173). Intracellularly, HSP90 acts as a core chaperone, working with co-chaperones to fold and mature client proteins. eHSP70 is released mainly from hepatocytes and lymphocytes via sympathetic activation of adrenergic receptors, while eHSP27 likely shares a release pathway with eHSP70/72 and originates from similar cell types under stress (174, 175).Moreover, therapeutic strategies must carefully modulate HSP activity to enhance protection while inhibiting deleterious effects, particularly HSP70 and HSP27.

The complex pathogenesis of DPN supports the exploration of combination therapies, such as HSP-targeting agents together with anti-inflammatory or antioxidant drugs, for potential synergistic effects. Translating this promise into viable treatments requires a rigorous translational pathway, commencing with robust validation in animal models of DPN and culminating in controlled clinical trials to confirm efficacy and safety.

Early-phase clinical trials should prioritize HSP-targeted agents with established human safety profiles, such as the approved HSP90 inhibitor TAS-116 (Pimitespib) (163, 176). Trials should prioritize the careful selection of patients, specifically those with early-stage and clinically manageable DPN. Thereby maximizing the potential to observe neuroprotective effects. Furthermore, trial endpoints must incorporate both objective measures (such as serial neurophysiological parameters, modified Toronto Clinical Neuropathy Score (mTCNS), intraepidermal nerve fibre density (IENFD), Michigan Neuropathy Screening Instrument Questionnaire and Examination (MNSIQ and MNSIE), etc.) and validated patient-reported outcomes for neuropathic pain and quality of life (177, 178). Concurrently, exploring biomarkers (such as HSP and cytokine levels) can provide crucial insights for target identification and efficacy assessment. Overcoming this translational challenge is therefore critical for the development of effective HSP-targeted interventions in DPN.

## 5 Conclusion

In conclusion, HSP play a central role in neuronal cellular responses to hyperglycaemic and oxidative stress, affecting protein homeostasis, mitochondrial function and inflammatory pathways. Treatments targeting them are expected to alter the course of DPN. Future studies should focus on elucidating the precise mechanisms of HSP-mediated neuroprotection, developing targeted drug delivery systems, and validating their stability as biomarkers.

## Glossary

**Table:**

DPN	Diabetic peripheral neuropathy
HSP	Heat shock protein
TAT-	HSP27 Trans-activator of transduction-Heat shock protein 27
T1DM	Type 1 diabetes
T2DM	Type 2 diabetes
AGEs	Advanced glycosylation end products
ERS	Endoplasmic reticulum stress
PKC	Protein kinase C
PARP	Poly ADP-ribose polymerase
AR	Aldose reductase
SDH	Sorbitol dehydrogenase
NADPH	Reduced Coenzyme II
ROS	Reactive oxygen species
RAGE	Advanced glycosylation end products receptor
NF-kB	Mammalian transcription factor NF-kB family pathway
IL-6	Interleukin 6
TNF-α	Tumour necrosis factor-alpha
3-DG	3-Deoxyglucuronide
ONOO^-^	Peroxynitrite
NO	Nitric oxide
iNOS	Inducible nitric oxide synthase
IRE1α-XBP1	Inositol-requiring enzyme 1α-X box-binding protein 1
CHOP	C/EBP homologous protein
ATP	Adenosine triphosphate
SC	Schwann cell
G-6-P	Glucose-6-phosphate
F-6-P	Fructose-6-Phosphate
G-3-P G	lyceraldehyde-3-phosphate
DAG	Diacylglycerol
Sp1	Specificity protein 1
O-GlcNAc	O-β-D-N-acetylglucosamine
TGF-β	Transforming growth factor-β
PAI-1	Plasminogen activator inhibitor-1
NAD+	Nicotinamide adenine dinucleotide
CRP	C-reactive protein
sICAM-1	Soluble intercellular adhesion molecule-1
COX-2	Cyclooxygenase-2
LPL	lipoprotein lipase
Rac1	GTP Ras-related C3 botulinum toxin substrate 1
SP	Stress protein
NTD	N-terminal structural domain
CTD	C-terminal structural domain
MD	Middle structural domain
GRP94	Endoplasmic reticulum glucose-regulated protein-94
TRAP1	Mitochondrial TNF receptor-associated protein 1
CDC37	Cell division cycle protein 37
Aha1	Activator of hsp90 ATPase activity 1
P23	Prostaglandin E synthase 3
CHORD	Cysteine- and histidine- rich structural domain
CRBN	Cereblon
TPR	Tetratricopeptide Repeat
MEEVD	Met-Glu-Glu-Val-Asp motif
FKBP36/38/51/52	Family of FK506-binding proteins
Toms	Mitochondrial outer membrane translocase
PP5	Protein phosphatase 5
HSC 70	Heat shock homologous protein 70
CHIP	CTD HSC70-responsive protein
USP19	Ubiquitin-specific protease 19
HOP	HSP70 and HSP90 histones
SGT	Glutamine-rich small TPR proteins
NBD	N-terminal nucleotide-binding structural domain
SBD	C-terminal substrate-binding structural domain
GRP78	Mitochondrial glucose-regulated protein-78
GRP75	Endoplasmic reticulum glucose-regulated protein-75
JDP	J-domain proteins
Bcl-2	B-cell lymphoma-2
BIM	Bcl-2 interaction mediator
BH3	Bcl-2 homology 3
ADP	Adenosine diphosphate
Pi	Free phosphate group
NEFs	Nucleotide exchange factors
BAG	Bcl-2-associated athanogene
AIPL1	Aryl hydrated carbon receptor interacting protein 1
BAG3	BAG cochaperone 3
HSE	Heat Shock Element
FTH1	Ferritin heavy chain 1
TfR	Transferrin receptor protein
iHSP27	Intracellular HSP27
eHSP27	Extracellular HSP27
HSF1	Heat shock factor 1
mtBE	Mitochondrial bioenergetics
MnSOD	Manganese superoxide dismutase
MRC	Maximum Respiratory Capacity
DMAG	Geldanamycin derivatives
iHSP70	Intracellular HSP70
eHSP70	Extracellular HSP70
mSOD1	Mutant superoxide dismutase 1
NOX2	NADPH oxidase 2
NOX4	NADPH oxidase 4
IkBα	NF-kB inhibitory protein α
IKK	IkB kinase
LPS	Lipopolysaccharide
JNK	c-Jun N-terminal kinase
CD14	LPS receptor
TLR2/4	Toll-like receptor 2/4
MHC-II	Major Histocompatibility Complex II
IL-1β	Interleukin 1β
NK	Natural killer cells
GSH	Glutathione
DRG	Dorsal root ganglion
T2DM-MCI	Type 2 diabetes mellitus with mild cognitive impairment
TMTA	Trail Making Test Part A
TMTB	Trail Making Test Part B
PPI	Protein-protein interaction
HSR	Heat shock reaction
HTT	Hot tub therapy
PQC	Protein quality control
JDM	J-domain mimic
GFP	Green fluorescent protein
GFPnb	Green fluorescent protein nano antibody
mCherry	Red fluorescent protein
cAMP	Cyclic adenosine monophosphate
PKA	Protein kinase A
mChnb	Red Fluorescent Protein nano Antibody
TSapphire	Blue fluorescent protein
Caspase	Cysteinyl asparaginase
SAH	Subarachnoid haemorrhage
CSF	Cerebrospinal fluid
HFD	High-fat diet
MKK4	Mitogen-activated protein kinase kinase 4
TAT-HSP27_65-90_	Trans-activator of transduction-Heat shock protein 27(amino acid residues 65-90)
CPP	Cell-penetrating peptide
Tfam	Mitochondrial transcription factor A
MPZ	Myelin protein zero
eHSP90α	Extracellular HSP90α
TAS-116	Pimitespib
mTCNS	modified Toronto Clinical Neuropathy Score
IENFD	Intraepidermal Nerve Fibre Density
MNSIQ	Michigan Neuropathy Screening Instrument Questionnaire
MNSIE	Michigan Neuropathy Screening Instrument Examinatio
